# Lipopolysaccharide triggers exacerbated microglial activation, excessive cytokine release and behavioural disturbances in mice with truncated Fused-in-Sarcoma Protein (FUS)

**DOI:** 10.1016/j.bbih.2023.100686

**Published:** 2023-09-15

**Authors:** Alexander Trofimov, Dmitrii Pavlov, Anand Goswami, Anna Gorlova, Kirill Chaprov, Aleksei Umriukhin, Allan Kalueff, Alexey Deykin, Klaus-Peter Lesch, Daniel Clive Anthony, Tatyana Strekalova

**Affiliations:** a Department of Psychiatry and Neuropsychology, School for Mental Health and Neuroscience, Maastricht University and Neuroplast BV, Maastricht, the Netherlands; b Hotchkiss Brain Institute, Alberta Children's Hospital Research Institute, University of Calgary, Calgary, Alberta, Canada; c Institute for Neuropathology, University Clinic RWTH Aachen, Germany; d Laboratory of Psychiatric Neurobiology, Institute of Molecular Medicine, Department of Normal Physiology, Sechenov First Moscow State Medical University, Russia; e Division of Pathophysiology (Biomedicine), School of Biosciences, Sir Martin Evans Building, Museum Avenue, Cardiff, CF10 3AX, Cardiff University, UK; f Institute of Translational Biomedicine, St. Petersburg State University, St. Petersburg, Russia; g Joint Center for Genetic Technologies and Department of Pharmacology and Clinical Pharmacology, Belgorod State National Research University, Belgorod, Russia; h Division of Molecular Psychiatry, Center of Mental Health, University Hospital of Würzburg, University of Würzburg, Germany; i Department of Pharmacology, University of Oxford, United Kingdom

**Keywords:** Frontotemporal lobar degeneration (FTLD), Amyotrophic lateral sclerosis (ALS), Fused in sarcoma (FUS) protein, Lipopolysaccharide (LPS), Interleukin-1β (IL-1β), Tumour necrosis factor (TNF), Cyclooxygenase-1 (COX-1), Cyclooxygenase-2 (COX-2), Emotionality

## Abstract

CNS inflammation, including microglial activation, in response to peripheral infections are known to contribute to the pathology of both familial and sporadic neurodegenerative disease. The relationship between Fused-in-Sarcoma Protein (FUS)-mediated disease in the transgenic FUS[1–359] animals and the systemic inflammatory response have not been explored. Here, we investigated microglial activation, inflammatory gene expression and the behavioural responses to lipopolysaccharide-induced (LPS; 0.1 mg/kg) systemic inflammation in the FUS[1–359] transgenic mice. The pathology of these mice recapitulates the key features of mutant FUS-associated familial frontotemporal lobar degeneration (FTLD) and amyotrophic lateral sclerosis (ALS). Here, pre-symptomatic 8-week-old mutant or wild type controls were challenged with LPS or with saline and sucrose intake, novel cage exploration, marble burying and swimming behaviours were analyzed. The level of pro-inflammatory gene expression was also determined, and microglial activation was evaluated. In chronic experiments, to discover whether the LPS challenge would affect the onset of ALS-like paralysis, animals were evaluated for clinical signs from 5 to 7 weeks post-injection. Compared to controls, acutely challenged FUS[1–359]-tg mice exhibited decreased sucrose intake and increased floating behaviours. The FUS[1–359]-tg mice exhibited an increase in immunoreactivity for Iba1-positive cells in the prefrontal cortex and ventral horn of the spinal cord, which was accompanied by increased expression of interleukin-1β, tumour necrosis factor, cyclooxygenase-(COX)-1 and COX-2. However, the single LPS challenge did not alter the time to development of paralysis in the FUS[1–359]-tg mice. Thus, while the acute inflammatory response was enhanced in the FUS mutant animals, it did not have a lasting impact on disease progression.

## 1 Introduction

Dysfunction of Fused in Sarcoma (FUS) gene expression is associated with frontotemporal lobar degeneration (FTLD) and sporadic and familial forms of amyotrophic lateral sclerosis (ALS) (Feldman et al., 2022; Puppala et al., 2021). Both FTDL and ALS are devastating incurable disorders and thus, their prevention and treatment remain an important unmet need (DeLoach et al., 2015; Sivasathiaseelan et al., 2019). Various forms of the FUS mutation cause RNA dysfunction and pathological protein aggregation in neurons, leading to their degeneration and death (Ling et al., 2013). Although the mechanism of cell death remains obscure.

The latest evidence also suggests that non-neuronal mechanisms, such as bystander damage following glial activation, may also contribute to FTDL and ALS pathology (Vahsen et al., 2021). For example abnormal recognition and responses to toxic elements, impaired phagocytosis, or a switch to a neurotoxic cytokine over-expression profile by dysfunctional microglia have all been shown to be associated with mutations of the genes associated with FDTL and ALS: SOD-1, TREM2, C9Orf72, GRN TBK1, OPTN, VCP, SQSTM1 and PFN1 (Feldman et al., 2022; McCauley and Baloh, 2019). Concerning FUS, transcriptome analysis of transgenic mice expressing the truncated highly-aggregate-prone form of human FUS has revealed abnormal expression of microglial genes (Funikov et al., 2018). In vitro, overexpression of wild-type FUS gene in mouse and human astrocytes has been reported to increase their sensitivity to the pro-inflammatory cytokine interleukin (IL)-1β leading to over-expression of inflammatory mediators, microglial activation, and neuronal cell death (Ajmone-Cat et al., 2019).

The mutations also result in systemic inflammation in FTLD and ALS patients, which is a prominent feature of these pathologies and contributes to their development (Goutman et al., 2022; McCauley and Baloh, 2019). Elevated levels of pro-inflammatory cytokines tumour necrosis factor (TNF), IL-6, granulocyte-macrophage colony-stimulating factor (G-CSF), macrophage inflammatory protein 1α (MIP1α) and other inflammatory mediators in the cerebrospinal fluid and blood were found to be elevated in FTLD and ALS patients (Bright et al., 2019; Hu et al., 2017; McCauley and Baloh, 2019).

The occurrence of systemic inflammation preceding FTLD or ALS has been recognized as a risk factor for these disorders. For example, bacterial and viral infections (Alam et al., 2017), occupational exposure to toxins of various kinds (Goutman et al., 2022; McCombe and Henderson, 2011), autoimmune conditions (Corzo et al., 2022; Lin et al., 2018; Sangha et al., 2020), e.g. type 1 diabetes, multiple sclerosis (Cui et al., 2021), and dysregulation of gut bacteria regulating inflammation (Nicholson et al., 2021) have all been shown the affect the development of FTLD or ALS. However, little is known about an interaction of pre-existing pro-inflammatory conditions with an aberrant immune response in genetically determined FTLD and ALS syndromes such as the FUS mutations. Here, we used the FUS[1–359]-tg mouse line, expressing a truncated highly-aggregate-prone form of human FUS, which displays FTLD-like and ALS-like disease halmarks in both pre-symptomatic and symptomatic stages at 8 and 14 weeks respectively (de Munter et al., 2020a, 2020b; Probert et al., 2022; Sambon et al., 2020; Shelkovnikova et al., 2013), in conjunction with a systemic lipopolysaccharide (LPS) challenge.

Earlier studies with the FUS[1–359]-tg mice revealed that the pathology is associated with over-expression of the pro-inflammatory cytokines IL-1β and IL-6 in the brain, blood and spinal cord, and behavioural deficits in the symptomatic FUS[1–359]-tg mice (de Munter et al., 2020a, 2020b; Probert et al., 2022; Lysikova et al., 2019). However, gene expression studies failed to reveal any signs of altered microglial function or of inflammatory markers in the CNS of naïve pre-symptomatic FUS[1–359]-tg mice (Chaprov et al., 2021). Here, we studied immunohistochemical expression of Iba-1 in the prefrontal cortex (PFC), hippocampus (HIP) and spinal cord (SC) of naïve pre-symptomatic 8-week old FUS[1–359]-tg mice 24 h after an LPS injection. We also measured gene expression for IL-1β, TNF, cyclooxygenase (COX)-1 and COX-2 at 24 and 48h following the challenge. In a separate cohort of mice, we investigated whether an LPS administration affects the onset of paralysis in the mutants from 5 to 7 weeks after the challenge.

## 2 Materials and methods

### 2.1 Animals and study design

**Fig. 1:**
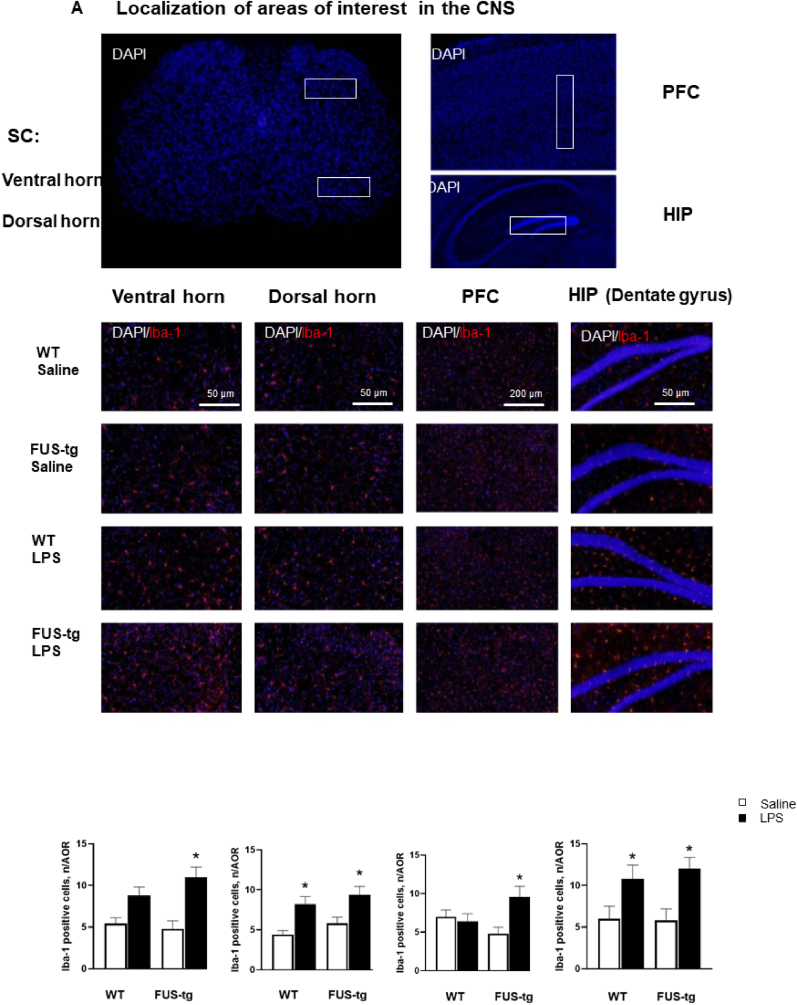
**Iba-1-staining in CNS of FUS-tg mice. (A)** Areas of interest in CNS. Representative image of Iba-1 and DAPI-staining in WT-saline, FUS-tg-saline, WT-LPS and FUS-tg-LPS mice. **(B)** LPS-induced increases of the density of Iba-1-positive cells in WT and FUS-tg mice (two-way ANOVA and Tukey's test; *p < 0.05 vs. respective control group).

8-week old FUS[1–359]-tg (FUS-tg) and wild type (WT) male mice were housed under standard conditions *(see Supplementary File (SF)*; all protocols complied with 2010/63/EU and ARRIVE guidelines (http://www.nc3rs.org.uk/arrive-guidelines). In the first set of experiments, ten mice of each genotype were assigned to four groups, and received an intraperitoneal (i.p.) injection of either saline or LPS (0.1 mg/kg; *see below*) and were tested for sucrose intake between 18 and 24h following the challenge and before being culled for an immunohistochemical (IHC) study of Iba-1-positive cell density in the PFC, HIP and SC 24h post-injection (Suppl.Fig.1A). In a second set of animals, 6–14 h following the same LPS-challenge, ten mutants and ten controls were investigated in the novel cage exploration test, pellet displacement behavior marble test and forced swim test and these animals were culled 24 h post-injection to determine the level of expression of selected pro-inflammatory mediators (*see below*) in the PFC, HIP and SC (Suppl.Fig.B). In the final cohort of animals, 8-week old FUS-tg mutants were injected with LPS or saline (12 and 13 animals per group, respectively) and the onset of paralysis after the injection was recorded (Sambon et al., 2020; Suppl. Fig.1C). Finally, ten 8-week-old mutants were assigned to the four groups, and were injected with either LPS or saline and culled for RT-PCR in selected CNS structures (Suppl.Fig.1D).

### 2.2 Behavioural tests

The behavioural experiments were performed as described elsewhere (de Munter et al., 2020a, 2020b; Probert et al., 2022; Strekalova and Steinbusch, 2010; Strekalova et al., 2022a, Strekalova et al., 2022b) (*see SF*).

### 2.3 LPS challenge

The mice (8-week-old FUS[1–359]-tg (FUS-tg) and wild type (WT) males) were injected with LPS dissolved in sterile saline (0.9%) (E.coli 0111:B6, Sigma, St. Louis, MO, USA) at the dose 0.1 mg/kg, i. p. In 0.1 mL of the sterile saline vehicle control (Couch et al., 2016; Trofimov et al., 2017).

### 2.4 Culling and tissue collection

Mice were terminally anaesthetized with an i. p. Injection of sodium pentobarbitone and their left ventricle was perfused with 10 mL ice-cold saline (for PCR assay) followed by a 4% paraformaldehyde solution (for IHC assay). The PFC, the HIP and lumbar segments of SC were isolated and stored as previously described (Couch et al., 2016; de Munter et al., 2020a).

### 2.5 Immunohistochemical analysis of iba-1-positive cells

**Fig. 2:**
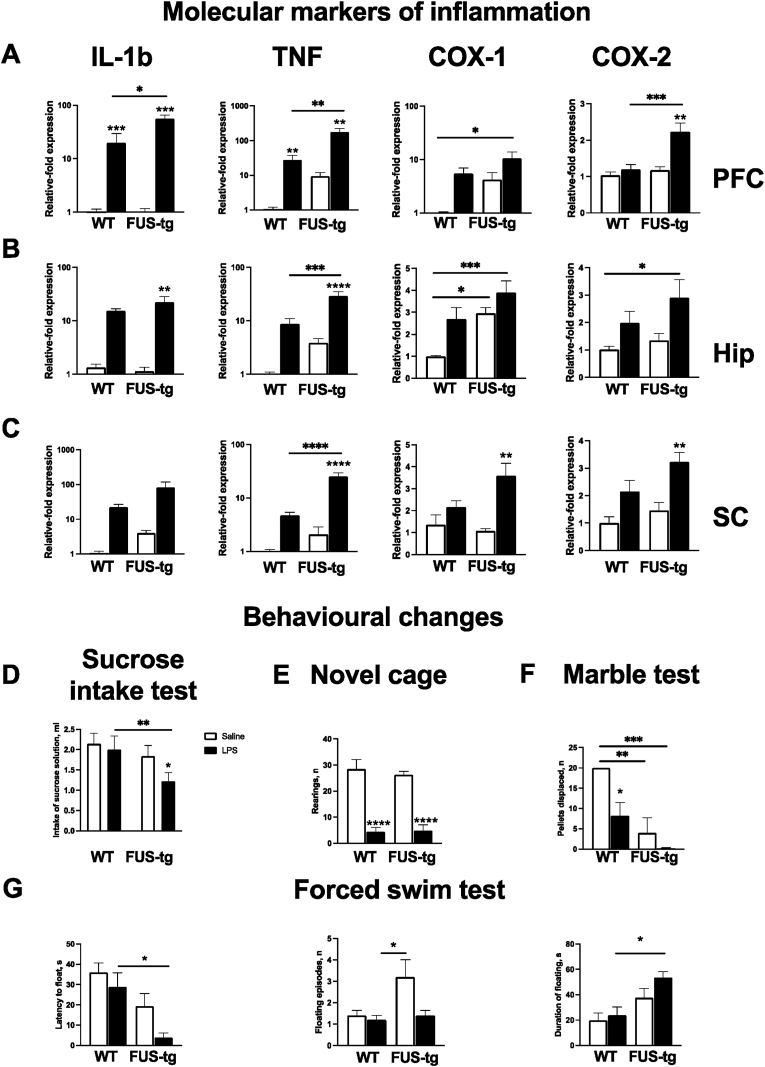
**LPS-induced molecular and behavioural changes in FUS-tg mice.** Following 24 h post-LPS-challenge, there were increases in mRNA of IL-1β, TNF, COX-1 and COX-2 in both genotypes, in **(A)** PFC, **(B)** HIP and **(C)** SC, where these changes were more pronounced in mutants. Note that COX-1 mRNA level was increased in Hip of FUS-tg mice prior to the LPS injection. Molecular changes were accompanied by **(D)** altered sucrose intake in FUS-tg-LPS group, **(E)** decreased total rearing scores in the novel cage and counted per minute in both genotypes, **(F)** lowered total number of displaced pellets in marble test and counted each 15 min in both genotypes, **(G)** shortened latency to float, prolonged duration of floating in mutants and unchanged number of floating episodes in FUS-tg-LPS group (two-way ANOVA and post-hoc Tukey's test: *p < 0.05; **p < 0.01; ***p < 0.001; ****p < 0.0001. Comparison without bar = saline versus LPS & bridging lines indicate other comparisons).

Immunostaining with Iba-1 antibodies and image analysis were performed as previously described (Couch et al., 2013; Strekalova et al., 2022a, Strekalova et al., 2022b) (*see SF*). Briefly, 10 μm-thick brain coronal sections and SC sections were cut, mounted on gelatin-coated slides, incubated with primary Iba-1 antibody and counter-stained with 4′,6-diamidino-2-phenylindole (DAPI). Cell counting was performed using ImageJ software and the density of Iba-1-positive cells was calculated. We also examined blood-brain barrier (BBB) breakdown in the 3 month-old animals at the point at which they were culled as they began to show clinical signs. There was no evidence of any BBB breakdown at this time point (Suppl.Fig.2).

### 2.6 Real-time polymerase chain reaction (RT-PCR)

mRNA was extracted by using TRI Reagent (Molecular Research Center, Inc., Cincinnati, OH, USA) from snap-frozen samples of PFC, HIP and SC (de Munter et al., 2020a, 2020b; Trofimov et al., 2017). First strand cDNA synthesis was performed using random primers and Superscript III transcriptase (Invitrogen, Darmstadt, Germany); 1 μg total RNA was converted into cDNA. RT-PCR of IL-1β, TNF, COX-1, COX-2 in the samples of PFC, HIP and SC were performed; relative gene expression was calculated using the ΔΔC_T_ method and normalized to the expression of glyceraldehyde 3-phosphate dehydrogenase (GAPDH) housekeeping gene and to the expression of the control sample (for list of primers, see *SF,* Table S1).

### 2.7 Statistics

Data were analyzed using GraphPad Prism version 9.0 for MacOS (San Diego, CA, USA). Two-way ANOVA followed by post-hoc Tukey tests were applied to analyze the effects of treatment in the two mouse genotypes. The level of significance was set p < 0.05. Data are Mean ± SEM.

## 3 Results

### 3.1 Iba-1-positive cell density in the CNS of FUS-tg mice challenged with LPS

A significant genotype × treatment interaction was discovered for Iba-1-positive cell density in the PFC, and a significant treatment effect was found in the ventral and dorsal horn of SC, and in the dentate gyrus of the HIP (p < 0.05, two-way ANOVA; *SF,* Table S2). While LPS-challenged control WT mice exhibited an increase in density of Iba-1 positive cells in the dorsal horn of the SC, the LPS-challenged FUS-tg animals exhibited increases in both dorsal and ventral horn of the SC, the PFC, and in the dentate gyrus of the HIP (Fig. 1A).

### 3.2 FUS-tg mice reveal increased baseline and LPS-induced cytokine expression 24h post-injection

In the prefrontal cortex (PFC) there was a significant genotype × treatment interaction for levels of expression of *IL-1β*, *TNF* and *COX-2*, as well as significant treatment and genotype effects; significant treatment effect was shown for *COX-1* expression (p < 0.05, two-way ANOVA; *SF,* Table S3). LPS-challenged FUS-tg mice showed significantly higher PFC expression of transcripts for the proinflammatory mediators (*IL-1β*, *TNF* and *COX-2*) than saline-treated FUS-tg mice and the LPS-challenged wild types (Fig. 2A). In the hippocampus (HIP), a significant genotype × treatment interaction for the concentration of *TNF* mRNA was found, and a significant treatment effect was shown for *IL-1β, TNF, COX-1* and *COX-2*. A significant genotype effect was found for *TNF* and *COX-1* (p < 0.05; *SF,* Table S4). Tukey post-hoc testing revealed significantly higher levels of *IL-1β* mRNA and *TNF* mRNA in FUS-tg-LPS-treated mice than in saline-treated FUS-tg animals; *TNF* was also higher in LPS-treated wild type mice compared to saline-treated controls. Saline vehicle-treated FUS-tg mice demonstrated an increased *COX-1* compared to saline vehicle-treated controls (Fig. 2B), which highly the presence of evolving inflammatory pathology at an early time point in the FUS mutants. In the SC, there was a significant genotype × treatment interaction for the concentrations of *TNF* mRNA, as well as significant treatment effect in concentrations of IL-1β mRNA, *TNF* mRNA, *COX-*1 mRNA and *COX-*2 mRNA and significant genotype effect for *TNF* mRNA and *COX-*2 mRNA (p < 0.05, *SF,* Table S5). Post-hoc testing revealed that there were significant increases in *TNF* mRNA, *COX-*1 mRNA and *COX-*2 mRNA levels in FUS-tg-LPS challenged mice in comparison with the saline-treated FUS-tg mice, as well as higher levels of *TNF* mRNA in the FUS-tg-LPS challenged mice than in LPS-challenged controls (Fig. 2C).

### 3.3 Exacerbated behavioural responses in LPS-challenged FUS-tg mice

A two-way ANOVA did reveal a genotype × treatment interaction for sucrose intake, but there was a significant effect of both the genotype and treatment on sucrose intake (p < 0.05, two-way ANOVA; *SF,* Table S6). Post-hoc testing revealed the presence of a significant decrease in sucrose intake in the LPS-challenged FUS-tg mice compared to both non-treated FUS-tg mice and LPS-challenged wild types (Fig. 2D). In the novel cage test, there was a significant effect of treatment on the number of rears (p < 0.05, *SF,* Table S6). A post-hoc Tukey test demonstrated a reduction of the number of rears in both LPS-treated groups in comparison with the saline-treated animals for each minute of observation and for the 5-min value (Fig. 2E). In the marble test, there was a significant effect of genotype and the treatment on the number of displaced pellets (p < 0.05, *SF,* Table S6). Post-hoc testing showed a reduced number of displaced pellets in LPS-challenged wild types and saline-treated FUS-tg mice both in comparison with saline-injected controls (Fig. 2F). In the forced swim test, there was a significant treatment effect on the latency to float, the number of floating episodes, but not on the duration of floating; a significant effect of genotype was found in first two parameters (p < 0.05, *SF,* Table S7). Tukey's post hoc test showed a significant reduction in the latency to float and an increase of the duration of floating in FUS-tg LPS-challenged mice in comparison with wild type LPS-challenged mice (Fig. 2G).

### 3.4 Lack of genotype differences in LPS-induced gene expression 48h post injection and unchanged onset of the ALS-like paralysis in LPS-challenged mutants

At time point 48h post-challenge, no genotype × treatment interaction differences were found in the mRNA concentrations of investigated genes (p > 0.05, two-way ANOVA; *SF,* Tables S8–10). There was a significant effect of treatment on this measure, no significant effect of genotype (p < 0.05 and p > 0.0,05, respectively); Tukey's test revealed no significant differences between LPS-treated groups (p < 0.05) suggesting a rapid decay of the exacerbated sensitivity to LPS administration of FUS-tg mice.

Finally, we found similar age in days at which the groups of LPS-challenged and unchallenged FUS-tg-mice displayed the first signs of paralysis after the injection (25.58 ± 2.11 and 25.38 ± 2.20, respectively, p = 0.93, *t-*test), which was at approximately 3 months of age.

## 4 Discussion

Following the LPS injection, two-way analysis of variance revealed suppression of hippocampus-dependent performance in the marble test and elevated gene expression of IL-1β, TNF, COX-1 and COX-2 in the hippocampus and spinal cord, and the response was always greater in the FUS-tg mice. It is noteworthy, that COX-1 mRNA was elevated in the hippocampus of FUS-tg mice prior to the LPS injection, indicating the presence of an evolving inflammatory process in the pre-symptomatic FUS-tg mice, which is in keeping with our previous data (de Munter et al., 2020b). Together, the data show that there is an exacerbated response to LPS in the young pre-symptomatic FUS-tg mice, and the effect is most obvious in the PFC. However, by 48h after the LPS injection, no genotype-associated differences in the immune response remained, suggesting a rapid decay of the increased pro-inflammatory response in the mutants. This may explain why the challenge failed to affect the onset of the ALS-like motor syndrome in FUS-tg mice subjected to a single LPS challenge found in our work.

Multiple genetic mutations are associated with familial forms of ALS, among which mutations in the FUS gene represent one subtype. The FUS[1–359] transgenic mouse model has been designed to carry a truncated form of the FUS protein to study the pathogenesis of ALS related to FUS mutations and the FUS[1–359] transgenic mouse model is primarily tailored to study FUS-mediated ALS pathogenesis. However, the insights gained here from this model are likely be relevant to understanding ALS from a broader perspective as the same molecular pathways are commonly disrupted in various genetic forms of ALS. For instance, mutant FUS can lead to mislocalization of the protein, and similar protein mislocalization or aggregation is observed in other ALS-related genes like TDP-43. Clearly, while there are shared pathways, each genetic mutation will also have its own unique pathology. It is important to note that the FUS[1–359] exhibits hallmark neuropathological features of ALS, such as motor neuron loss, protein aggregates, and gliosis. Even if the FUS[1–359] model primarily represents FUS-related pathology, potential treatments that prove effective in this model may have broader applicability. Conversely, treatments targeting very specific FUS mechanisms might not be relevant for other forms of ALS, but the insights gained are important for those with FUS mutations. As there is growing interest in understanding how various ALS-related genes might interact with other challenges, and here we sought to explore the interaction between a pro-inflammatory challenge (LPS) and the FUS mutation. The LPS challenge is a somewhat reductionist approach to mimicking a bacterial infection, which would be expected to last for much longer, but our experiments reveal that the brain of the FUS-tg mouse is primed and is more susceptible to such systemic inflammatory challenges and gave rise to elevated production of cytokines and sickness behaviors. We employed a LPS challenge of 0.1 mg/kg, that is not considered sufficient to cause BBB breakdown or give rise to long-lasting pathological or behavioural changes. We have previously shown that the administration of 0.5 mg/kg LPS, which was used to reactivate a quiescent MS-like lesion in the murine brain, does not cause any BBB breakdown despite increasing local rCBV and provoking new leukocyte recruitment to the lesion (Serres et al., 2009). Others have used 3 mg/kg to increase the permeability of the BBB, which induces sepsis like conditions in rodent (Xaio et al., 2001).

We accept that the addition of the FUS pathology with a low dose of LPS used here might have a synergistic effect on blood-brain barrier integrity, a factor that might contribute to elevated cytokine production in the brain, and we will investigate this possibility in future studies. However, we chose the 0.1 mg/kg dose to try avoid such complications. It is of note that we have recently shown that LPS is able to gain access across an intact BBB by piggybacking on HDL, and thus the BBB may not represent the barrier that it is perceived to be in for such a lipophilic compound as LPS is known to be (Radford-Smith et al., 2023). Indeed, while our observations highlight the prominent role of neuro- and systemic inflammation observed by many in ALS/FTLD (McCauley and Baloh, 2019), they may also account for why therapeutic approaches to decrease inflammation have thus far failed to alter disease course in humans. Insomuch that if anti-inflammatory, antiviral, or antibiotic therapy is to be trialed, it should be timed with infections/injuries rather than as a generalized ongoing treatment regimen.

The rapid increase in Iba-1 staining in the PFC is most likely to represent activation rather than proliferation and was accompanied by depressive-like behaviors which are held to be largely regulated by this brain structure, where such behaviors can be induced by pro-inflammatory cytokine administration (Hayley et al., 2013). The deficits in the marble test and increased floating behavior in naive mutants are likely to be associated with increased baseline TNF and COX-1 expression in the PFC and HIP (Puppala et al., 2021). The suppression of exploratory novel cage activity in both genotypes after LPS challenge might be explained by the short delay between the inflammatory challenge and the assay, and a ceiling pro-inflammatory effects on mouse behavior (Schapovalova et al., 2022; Yates et al., 2022). The lack of these group differences also rules out possible confounds in measuring reported LPS-induced behavioural responses of two genotypes in other tests, which might have, potentially, been caused by distinct genotype changes in general activity after LPS injection.

## 5 Conclusions

Our study has revealed the presence of an increased sensitivity of microglia to inflammatory challenges at in early pre-symptomatic stages of the ALS/FTLD-like syndrome in mice with compromised FUS function. It is accompanied by elevated CNS cytokine production under unchallenged conditions and is present prior any signs of neurodegeneration in FUS-tg mutants (de Munter et al., 2020b; Chaprov et al., 2021), thus suggesting these pro-inflammatory processes are independent of neuronal cell death. Finally, the FUS[1–359]-tg mouse line used here can be a useful model to address the role of microglia and inflammation in the mechanisms of ALS/FTLD syndrome further.

## Declaration of competing interest

None of the authors involved in the work have any competing interests. We confirm that this manuscript has not been published elsewhere and is not under consideration by another journal.

## Data Availability

Data, which was all collected prior to 24 February 2022, will be made available on request.
