# Supplementary material for: Lipopolysaccharide triggers exacerbated microglial activation, excessive cytokine release and behavioural disturbances in mice with truncated Fused-in-Sarcoma Protein (FUS)
Source: Brain Behav Immun Health. 2023 Sep 15;33:100686. doi: 10.1016/j.bbih.2023.100686 (PMC10520340; doi:10.1016/j.bbih.2023.100686)
Supplement: Multimedia component 1 [file mmc1.docx]

**Supplementary File**

**Animals and housing conditions**

Mouse colony of FUS[1-359]-tg (FUS-tg) mice and their wild type littermates (WT) were bred and housed in the FDA-certified SPF facilities of the IPAC Center of Pre-clinical Trials (http://www.spf-animals.ru/about/providers/animals). 8-weeks-old male mice of both genotypes were single housed in standard plastic cages (27x22x15) and maintained on a 12-hour light/dark cycle (lights on at 21:00), under controllable laboratory conditions (22 ± 1°C, 55% humidity, room temperature 22-24ºC), food and water were available ad libitum. Experimental procedures were set up in accordance with a Directive 2010/63/EU and ARRIVE guidelines (<https://www.nc3rs.org.uk/arrive-animal-research-reporting-vivo-experiments>) and approved by the local veterinarian Committee for Bioethics of IPAC (N19‐16.06.2017) and MSMU (22/10/17‐MSMU‐35). All efforts were undertaken to ensure compliance with above‐mentioned regulations concerning human endpoint in animal research.

**Generation of FUS Transgenic Mice**

The generation of FUS-tg mice was performed as describe elsewhere (Robinson et al., 2015). Briefly, a fragment of human FUS[1–359] cDNA including 9 bp of 5′-UTR was cloned into Thy-1 promoter plasmid 323-pTSC21k. A gel-purified fragment obtained by digestion of the resulting plasmid DNA with NotI was used for microinjection of mouse oocytes. Transgenic animals were identified by PCR analysis of DNA from ear biopsies by the presence of 255-bp product (primers 5′-TCTTTGTGCAAGGCCTGGGT-3′ and 5′-AGAAGCAAGACCTCTGCAGAG-3′). Originally produced transgenic line on C57Bl6/CBA genetic background was backcrossed with CD1 wild type mice by several (>7) generations.


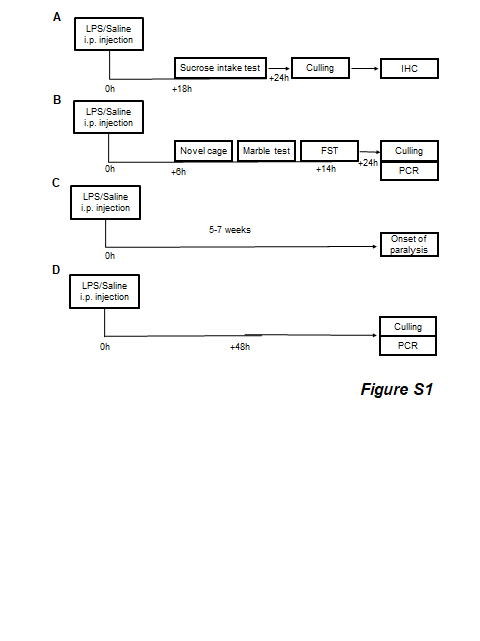


**Supplementary Figure 1: Experimental design.** Study **(A)** was performed to examine sucrose preference and the immunohistochemical investigation of Iba-1- and neuronal numbers in the CNS of saline and LPS-challenged mice. Study **(B)** comprised of behavioural tests and RT-PCR for inflammatory gene expression in the CNS saline and LPS-challenged mice at 24 h post-injection. Study **(C)** involved assessment of the onset of ALS-like paralysis in the LPS or saline-injected mutants. In study **(D)** we investigated, by RT-PCR, inflammatory gene expression in the CNS of saline and LPS-challenged mice at 48 h post-injection. **IHC**- immunohistochemistry study, **FST** – forced swim test, **LPS** – lipopolysaccharide.

**General conditions of behavioural studies**

Experiments were performed during the dark period of light cycle, followed by offline analysis. The person performing the experiments was blind for the genotype and treatment until the end of the behavioral tests. Experiments were carried out in the same rooms by the same persons between 09:00 and 17:00 h. Animals were allowed to adapt to experimental room for at least 1 h before testing.

*Sucrose test*

Mice were given 6 hours of free choice between two bottles of either 1% sucrose or standard drinking water, as described elsewhere (Strekalova et al., 2022). Bottles were weighed before and after conducting the sucrose intake test, and consumption calculated accordingly. The beginning of the test started with the onset of the dark (active) phase of animals’ cycle. To prevent the possible effects of side preference in drinking behaviour, the position of the bottles in the cage was switched at 3 hours, halfway through testing. No previous food or water deprivation was applied before the test. Other conditions of the test were applied as described elsewhere (Strekalova and Steinbusch 2010).

*Novel Cage Test*

The 5-min long novel cage test was carried out to assess exploration of a new environment as described elsewhere (Strekalova et al., 2004; Couch et al., 2016; Veniaminova et al., 2020). Mice were introduced into a standard plastic cage (21 cm x 21 cm x 15 cm) filled with fresh sawdust. The number of exploratory rears was counted under red light per each minute, and summed up for minutes 1-5 of the test.

*Pellet displacement marble test*

All experimental groups were tested for pellet displacement in a marble test as described elsewhere (Strekalova and Steinbusch 2010; Veniaminova et al., 2017, 2020). A tendency to displace small objects, e.g. small stones or food pellets, from a tube inside the cage, is species-specific in mice and has been demonstrated to depend on an intact hippocampal formation. Using a paper tube (internal diameter 4 cm, length 10 cm), filled with 20 food pellets and placed in the middle of a home cage (21 cm × 27 cm × 14 cm), the number of food pellet displaced by each mouse was assessed every 15 min during 1 h and 45 min.

*Forced Swim Test*

The Porsolt forced swim test (FST) was used as described elsewhere ([Strekalova et al., 2011](#_ENREF_31); Malatynska et al., 2012). Mice were subjected to a 6-min swimming session in a transparent cylinder (Ø 17 cm) filled with water (+23 °C, water height 13 cm, height of cylinder 20 cm, illumination intensity 25 Lux). Floating behaviour was defined by the absence of any directed movements of the animals’ head and body and was scored off-line. Using this method, the latency of the first episode of floating, the number of floating episodes and the duration of floating behaviour were recorded. Latency to begin floating was scored as time between introduction of the animal into the pool and the first moment of complete immobility of the entire body for a duration of >3 seconds. The total time spent floating was scored for the entire duration of the test using post-test video footage.

**Immunohistochemical analysis of Iba-1 -positive cells**

Immunostaining with Iba-1 and DAPI-staining and image analysis in the CNS of mice were performed as described elsewhere (Couch et al., 2016; Veniaminova et al., 2020). Coronal 10 μm-thick sections were cut on a cryostat microtome (Leica Biosystems, Nussloch, Germany) and mounted on gelatin-coated slides. Prefrontal sections were taken between 1.4 and 2.8 anterior-posterior axis; hippocampal sections were taken from lateral 3.6 to lateral 0.4 mm along the medial-lateral axis up to bregma (Paxinos and Franklin, 2001). Spinal cord sections were taken from the lumbar part. Slides were washed in PBS for 30 min and blocked for non-specific protein binding with 10% goat serum in PBS for 1 hour. Then, sections were incubated with primary antibody (Iba-1: 1:800, ab5076, Abcam, Cambridge, UK) in 1% normal goat serum at 4°C for 12 h. Visualization was performed using secondary antibodies anti-chicken-Alexa Fluor 647 (1:500, ThermoFisher, Abingdon, UK) in 1% serum in PBS (Vector Laboratories, Burlingame, CA, USA) for four hours at room temperature. To visualize the nuclei of the cells, sections were co-stained with 4′,6-diamidino-2-phenylindole (DAPI) (Santa Cruz Biotechnology, Santa Cruz, CA, USA). Immunostaining of ventral and dorsal horns of lumbar part of spinal cord (SC), prefrontal cortex (PFC) and dentate gyrus of the hippocampus (HIP) was examined using a microscope Leitz Dialux 20 (Leica, Wetzlar, Germany) and digital camera Basler ACE (Basler Group, Ahrensburg, Germany). These areas were specifically delineated according to the Paxinos atlas. Cell counting was carried out using ImageJ software. Three sections per each structure per animal were analyzed.

**Quantitative RT-PCR (qPCR)**

RNA extraction was performed as previously described from specifically microdissected snap-frozen brain regions and lumbar section of spinal cord ([Couch et al., 2016](#_ENREF_9), de Munter et al., 2020a,b). mRNA was extracted by using TRI Reagent (Molecular Research Center, Inc., Cincinnati, OH, USA). First strand cDNA synthesis was performed using random primers and Superscript III transcriptase (Invitrogen, Darmstadt, Germany); 1 μg total RNA was converted into cDNA. Standard curves were generated using total cDNA to enable normalization to three housekeeping genes glyceraldehyde-3-phosphate (GAPDH), TATA-binding-protein (Tbp), and beta-Actin (ActB). The latter two genes were excluded due to their less stable expression than that of GAPDH. qPCR was performed using the SYBR Green master mix (Bio-Rad Laboratories, Philadelphia, PA, USA) and the CFX96 Real-time System (Bio-Rad Laboratories, Philadelphia, , USA) for IL-1 beta, TNF, COX1, COX2 genes. Details of primers can be found in below (*Table 1*). Data were calculated as relative-fold changes compared to control mice as described elsewhere (Couch et al., 2016). Results of qRT-PCR measurement were expressed as Ct values, where Ct is defined as the threshold cycle of PCR at which amplified product was 0.05% of normalized maximal signal. We used the comparative Ct method and computed the difference between the expression of the gene of interest and the expression of housekeeping gene GAPDH in each cDNA sample (2-ΔΔ Ct method). Data are given as expression-folds compared to the mean expression values in control mice. Results are expressed as relative-fold compared to control animals.

***Table S1. Sequences of primers used***

| **Gene** | **Forward primer 5′–3′** | **Reverse primer 5′–3′** |
| --- | --- | --- |
| *GAPDH* | TGCACCACCAACTGCTTAG | GGATGCAGGGATGATGTTC |
| *IL-1β* | AACCTGCTGGTGTGTGACGTTTC | CAGCACGAGGCTTTTTTGTTGT |
| *TNF* | GCCTGTAGCCCACGTCGTA | GGCACCACTAGTTGGTTGTCTTTG |
| *COX-1* | GCCTGAGCCCAGATATAGCA | TTTCCGGCTAGAGGTGGGTA |
| *COX-2* | CCGTGCTGCTCTGTCTTAAC | TTGGGAACCCTTCTTTGTTC |

**Iba-1-positive cell density in the CNS of mice challenged with LPS**

Iba-1-positive cell density was increased in all investigated brain structures of LPS-challenged FUS-tg animals.

**Table S2. Summary of genotype and treatment effects and group comparisons in the Iba-1-positive cell density in the CNS.** 2-way ANOVA revealed significant genotype x treatment interaction for the density of Iba-1-positive cells in the PFC (F_1,16_=6.688, p=0.0199), but not for the ventral (F_1,16_=1.941, p=0.1827) and dorsal horn of SC (F_1,16_=0.0137, p=0.9080) and the dentate gyrus of the HIP (F_1,16_=0.2207, p=0.6448). There was a significant treatment effect in the ventral (F_1,16_=22.81, p=0.0002) and dorsal horn of SC (F_1,16_=18.88, p=0.0005), and in the dentate gyrus of the HIP (F_1,16_=13.63, p=0.0020), but not PFC (F_1,16_=4.046, p=0.0614). No significant genotype effect was found for density of Iba-1-positive cells in the ventral (F_1,16_=0.6337, p=0.4377) and dorsal horn of SC (F_1,16_=2.331, p=0.1463), PFC (F_1,16_=0.229, p=0.639), and dentate gyrus of the HIP (F_1,16_=0.1126, p=0.7415). Tukey analysis revealed significant group differences (**in bold**); ns: not significant. Sal: saline, LPS: lipopolysaccharide, increase of Iba-1-positive cell density.

| **Groups** | **Ventral horn of SC** | **Dorsal horn of SC** | **PFC** | **Dentate gyrus of the HIP** |
| --- | --- | --- | --- | --- |
| **WT-Sal vs FUS-tg-Sal** | p=0.974, ns | p=0.658, ns | p=0.466, ns | p=0.999, ns |
| **WT-LPS vs FUS-tg-LPS** | p=0.434, ns | p=0.754, ns | p=0.145, ns | p=0.939, ns |
| **WT-Sal vs -WT LPS** | p=0.119, ns | **p=0.028** | p=0.977, ns | p=0.145, ns |
| **FUS-tg-Sal vs FUS-tg-LPS** | **p=0.025** | **p=0.039** | **p=0.0233** | **p=0.0427** |

**Increased LPS-induced cytokine expression 24 h post injection**

At time point 24 h post-challenge, mRNA concentrations of investigated cytokines were increased in CNS structure of LPS-challenged FUS-tg mice.

**Table S3. Summary of genotype and treatment effects and group comparisons in 24h post-injection gene expression of inflammatory markers in the PFC.** There was a significant genotype x treatment interaction in mRNA concentrations of IL-1β (F_1,33_=4.927, p=0.0334), TNF (F_1,33_=4.593, p=0.0396) and COX-2 (F_1,33_=5.865, p=0.0211), but not COX-1 (F_1,31_=0.1674, p=0.6852). Significant genotype effect was found for IL-1β (F_1,33_=4.931, p=0.0334), TNF (F_1,33_=5.75, p=0.0223) and COX-2 (F_1,33_=10.17, p=0.0031), but not COX-1 (F_1,31_=3.524, p=0.0699) Significant treatment effect was revealed for IL-1β (F_1,33_=20.48, p<0.0001), TNF (F_1,33_=8.691, p=0.0058), COX-1 (F_1,31_=5.995, p=0.0202) and COX-2 (F_1,33_=10.85, p=0.0024. Tukey analysis revealed significant group differences (**in bold**); ns: not significant. Sal: saline, LPS: lipopolysaccharide, - increase of gene expression.

| **Groups** | **IL-1β** | **TNF** | **COX-1** | **COX-2** |
| --- | --- | --- | --- | --- |
| **WT-Sal vs FUS-tg-Sal** | p>0.999, ns | p=0.998, ns | p=0.752, ns | p=0.956, ns |
| **WT-LPS vs FUS-tg-LPS** | **p=0.0103** | **p=0.084** | p=0.346, ns | **p=0.0009** |
| **WT-Sal vs -WT LPS** | p=0.402, ns | p=0.954, ns | p=0.494, ns | p=0.932, ns |
| **FUS-tg-Sal vs FUS-tg-LPS** | **p=0.0001** | **p=0.0039** | p=0.194, ns | **p=0.0011** |

**Table S4. Summary of genotype and treatment effects and group comparisons in 24h post-injection gene expression of inflammatory markers in the HIP.** 2-way ANOVA revealed significant genotype x treatment interaction in mRNA concentration of TNF (F_1,32_=6.473, p=0.016), but not IL-1β (F_1,33_=0.7142, p=0.04041), COX-1 (F_1,31_=0.7245, p=0.4012), and COX-2 (F_1,33_=0.3408, p=0.5633). Significant treatment effects were revealed in mRNA concentration of IL-1β (F_1,33_=15.59, p=0.0003), TNF (F_1,32_=23.29, p<0.0001), COX-1 (F_1,31_=8.9, p=0.0055), and COX-2 (F_1,33_=6.217, p=0.0178). Significant genotype effect was shown in mRNA concentration of TNF (F_1,32_=11.59, p=0.0018), COX-1 (F_1,31_=12.77, p=0.0012) but not IL-1β (F_1,33_=0.636, p=0.431), and COX-2 (F_1,32_=1.524, p=0.2257). Tukey analysis revealed significant group differences (**in bold**); ns: not significant. Sal: saline, LPS: lipopolysaccharide, - increase

of gene expression

| **Groups** | **IL-1β** | **TNF** | **COX-1** | **COX-2** |
| --- | --- | --- | --- | --- |
| **WT-Sal vs FUS-tg-Sal** | p>0.999, ns | p=0.934 | **p=0.0246** | p=0.972, ns |
| **WT-LPS vs FUS-tg-LPS** | p=0.605, ns | **p=0.0008** | p=0.209, ns | p=0.524, ns |
| **WT-Sal vs -WT LPS** | **p=0.014** | p=0.409, ns | p=0.0619, ns | p=0.563, ns |
| **FUS-tg-Sal vs FUS-tg-LPS** | **p=0.0055** | **p<0.0001** | p=0.4122, ns | p=0.132, ns |

**Table S5. Summary of genotype and treatment effects and group comparisons in 24h post-injection gene expression of inflammatory markers in the SC.** There was a significant genotype x treatment interaction in mRNA concentrations of TNF (F_1,33_=11.49, p=0.0018), but not IL-1β (F_1,34_=1.524, p=0.2254), COX-1 (F_1,33_=3.648, p=0.0649), and COX-2 (F_1,33_=0.8148, p=0.3733). Significant treatment effect was shown in mRNA concentration of IL-1β (F_1,34_=4.587, p=0.0395), TNF (F_1,33_=22.05, p<0.0001), COX-1 (F_1,33_=13.41, p=0.0009), and COX-2 (F_1,33_=17.91, p=0.0002). Significant genotype effect was revealed in mRNA concentration of TNF (F_1,33_=14.12, p=0.0007) and COX-2 (F_1,33_=4.909, p=0.0337) ,but not IL-1β (F_1,34_=1.845, p=0.1833), and COX-1 (F_1,33_=1.594, p=0.2156) Tukey analysis revealed significant group differences (**in bold**); ns: not significant. Sal: saline, LPS: lipopolysaccharide, - increase of gene expression.

| **Groups** | **IL-1β** | **TNF** | **COX-1** | **COX-2** |
| --- | --- | --- | --- | --- |
| **WT-Sal vs FUS-tg-Sal** | p=0.998, ns | p=0.995, ns | p=0.973, ns | p=0.818, ns |
| **WT-LPS vs FUS-tg-LPS** | p=0.211, ns | **p<0.0001** | p=0.0972, ns | p=0.106, ns |
| **WT-Sal vs -WT LPS** | p=0.922, ns | p=0.807, ns | p=0.63, ns | p=0.123, ns |
| **FUS-tg-Sal vs FUS-tg-LPS** | p=0.089, ns | **p<0.0001** | **p=0.015** | **p=0.0036** |

**Exacerbated behavioural responses in LPS-challenged FUS-tg mice**

LPS-challenged FUS-tg mice demonstrated decreased sucrose intake, explorative behaviour in the novel cage and marble test, as well as prolonged floating in the forced swim test.

**Table S6. Summary of genotype and treatment effects and group comparisons in behavioural responses to LPS the in sucrose test, novel cage and marble test.** 2-way ANOVA revealed a significant effect of the genotype (F_1,16_=19.25, p=0.0005) and treatment (F_1,16_=9.531, p=0.0071) on the sucrose intake, but no significant effect of their interaction (F_1,16_=3.802, p=0.0689). In the novel cage test, significant effect of the treatment was found in the number of rearings (F_1,16_=88.28, p=0.0001), but no significant effect of genotype (F_1,16_=0.1385, p=0.7147) or genotype x treatment interaction (F_1,16_=0.2889, p=0.5983). In the marble test, significant effect of genotype (F_1,16_=23.53, p=0.0002) and the treatment (F_1,16_=9.941, p=0.0062) in the number of displaced pellets were demonstrated, but no significant effect of their interaction (F_1,16_=2.614, p=0.1254). Tukey analysis revealed significant group differences (**in bold**); ns: not significant. Sal: saline, LPS: lipopolysaccharide, - decrease of behavioral score.

| **Groups** | **Sucrose intake** | **Novel cage** | **Marble test** |
| --- | --- | --- | --- |
| **WT-Sal vs FUS-tg-Sal** | p=0.344, ns | p=0.916, ns | **p=0.0016** |
| **WT-LPS vs FUS-tg-LPS** | **p=0.0019** | p=0.999, ns | p=0.143, ns |
| **WT-Sal vs -WT LPS** | p=0.852, ns | **p<0.0001** | **p=0.0183** |
| **FUS-tg-Sal vs FUS-tg-LPS** | **p=0.0125** | **p<0.0001** | p=0.703, ns |

**Table S7. Summary of genotype and treatment effects and group comparisons behavioural responses to LPS in the forced swim test scores in LPS-challenged FUS-tg mice.** 2-way ANOVA revealed no significant treatment x genotype interaction in the latency of floating (F_1,16_=0.6144, p=0.4446), the number of floating episodes (F_1,16_=3.200, p=0.0926) and the duration of floating (F_1,16_=0.9357, p=0.3478). A significant effect of genotype was found in the latency of floating (F_1,16_=15.07, p=0.0013), the number of floating episodes (F_1,16_=5.000, p=0.0399) and the duration of floating (F_1,16_=14.72, p=0.0015). There was a treatment effect on the latency to float (F_1,16_=4.527, p=0.0493), the number of floating episodes (F_1,16_=5.000, p=0.0399), but not the duration of floating (F_1,16_=2.599, p=0.1265). Tukey analysis revealed significant group differences (**in bold**); ns: not significant. Sal: saline, LPS: lipopolysaccharide,

- increase of behavioral score, - decrease of behavioral score.

| **Groups** | **Latency of floating** | **Number of floating episodes** | **Duration of floating** |
| --- | --- | --- | --- |
| **WT-Sal vs FUS-tg-Sal** | p=0.168, ns | p=0.515, ns | p=0.219, ns |
| **WT-LPS vs FUS-tg-LPS** | **p=0.0212** | p=0.989, ns | **p=0.0174** |
| **WT-Sal vs -WT LPS** | p=0.778, ns | p=0.989, ns | p=0.967, ns |
| **FUS-tg-Sal vs FUS-tg-LPS** | p=0.209, ns | p=0.515, ns | p=0.299, ns |

**Lack of genotype differences in LPS-induced cytokine expression 48 h post injection**

At time point 48 h post-challenge, no genotype differences were found in the mRNA concentrations of investigated cytokines in any of investigated CNS structures

**Table S8. Summary of genotype and treatment effects and group comparisons in 48 h post-injection gene expression of inflammatory markers in the PFC.** 2-way ANOVA revealed no significant genotype x treatment interaction for mRNA expression of IL-1β (F_1,16_=0.3172, p=0.1412), TNF (F_1,16_=3.069, p=0.1192), COX-1 (F_1,16_=0.1002, p=0.1569) and COX-2 (F_1,16_=1.4923, p=0.7244). There was no significant genotype effect for mRNA expression of IL-1β (F_1,16_=0.2731, p=0.9729), TNF (F_1,16_=1.969, p=0.3418), COX-1 (F_1,16_=0.9253, p=0.1725) and COX-2 (F_1,16_=0.1239, p=0.9212). There was no significant treatment effect for mRNA expression of IL-1β (F_1,16_=0.7823, p=0.1238), TNF (F_1,16_=2.761, p=0.3316), COX-1 (F_1,16_=0.1909, p=0.1333) and COX-2 (F_1,16_=0.7319, p=0.092). Tukey analysis revealed significant group differences in target molecules (**in bold**); ns: not significant. Sal: saline, LPS: lipopolysaccharide, - increase of gene expression.

| **Groups** | **IL-1β** | **TNF** | **COX-1** | **COX-2** |
| --- | --- | --- | --- | --- |
| **WT-Sal vs FUS-tg-Sal** | p=0.515, ns | p=0.342, ns | p=0.115, ns | p=0.216, ns |
| **WT-LPS vs FUS-tg-LPS** | p=0.301, ns | p=0.629, ns | p=0.449 ns | p=0.545 ns |
| **WT-Sal vs -WT LPS** | **p=0.001** | **p=0.03** | p=0.431, ns | p=0.602, ns |
| **FUS-tg-Sal vs FUS-tg-LPS** | **p=0.001** | p=0.565, ns | p=0.404, ns | p=0.328, ns |

**Table S9.** **Summary of** **genotype and treatment effects and group comparisons in 48 h post-injection gene expression of inflammatory markers in the PFC.** 2-way ANOVA revealed no significant genotype x treatment interaction for mRNA expression of IL-1β (F_1,16_=0.5129, p=0.0041), TNF (F_1,16_=2.7926, p=0.1256), COX-1 (F_1,16_=0.1907, p=0.1334) and COX-2 (F_1,16_=0.8921, p=0.2406). There was no significant genotype effect for mRNA expression of IL-1β (F_1,16_=0.5418, p=0.2345), TNF (F_1,16_=2.9025, p=0.2759), COX-1 (F_1,16_=0.9229, p=0.9193) and COX-2 (F_1,16_=0.4519, p=0.2209). There was no significant treatment effect for mRNA expression of IL-1β (F_1,16_=1.1816, p=0.7189), TNF (F_1,16_=1.721, p=0.2725), COX-1 (F_1,16_=0.1972, p=0.1193) and COX-2 (F_1,16_=0.9087, p=0.5644). Tukey analysis revealed significant group differences in target molecules (**in bold**); ns: not significant. Sal: saline, LPS: lipopolysaccharide, - increase of gene expression.

| **Groups** | **IL-1β** | **TNF** | **COX-1** | **COX-2** |
| --- | --- | --- | --- | --- |
| **WT-Sal vs FUS-tg-Sal** | p=0.605, ns | p=0.391, ns | p=0.218, ns | p=0.5776, ns |
| **WT-LPS vs FUS-tg-LPS** | p=0.311, ns | p=0.132, ns | p=0.116, ns | p=0.392 ns |
| **WT-Sal vs -WT LPS** | **p=0.03** | p=0.427, ns | p=0.374, ns | p=0.460, ns |
| **FUS-tg-Sal vs FUS-tg-LPS** | **p=0.001** | p=0.219, ns | p=0.692, ns | p=0.531, ns |

**Table S10.** **Summary of** **genotype and treatment effects and group comparisons in 48h post-injection gene expression of inflammatory markers in the SC.** 2-way ANOVA revealed no significant genotype x treatment interaction for mRNA expression of IL-1β (F_1,16_=0.5073, p=0.7142), TNF (F_1,16_=2.746, p=0.1132), COX-1 (F_1,16_=0.1982, p=0.1199) and COX-2 (F_1,16_=0.6439, p=0.2904). There was no significant genotype effect for mRNA expression of IL-1β (F_1,16_=0.5331, p=0.2109), TNF (F_1,16_=2.026, p=0.2119), COX-1 (F_1,16_=0.2963, p=0.9123) and COX-2 (F_1,16_=0.4939, p=0.2402). There was no significant treatment effect for mRNA expression of IL-1β (F_1,16_=0.7013, p=0.2182), TNF (F_1,16_=2.761, p=0.3292), COX-1 (F_1,16_=0.1012, p=0.1563) and COX-2 (F_1,16_=0.6909, p=0.4742). Tukey analysis revealed significant group differences in target molecules (**in bold**); ns: not significant. Sal: saline, LPS: lipopolysaccharide.

| **Groups** | **IL-1β** | **TNF** | **COX-1** | **COX-2** |
| --- | --- | --- | --- | --- |
| **WT-Sal vs FUS-tg-Sal** | p=0.518, ns | p=0.318, ns | p=0.682, ns | p=0.724 ns |
| **WT-LPS vs FUS-tg-LPS** | p=0.413, ns | p=0.522, ns | p=0.528, ns | p=0.462 ns |
| **WT-Sal vs -WT LPS** | p=0.397, ns | p=0.372, ns | p=0.334, ns | p=0.549, ns |
| **FUS-tg-Sal vs FUS-tg-LPS** | p=0.439, ns | p=0.312, ns | p=0.416, ns | p=0.295, ns |

**Supplementary Figure 2**


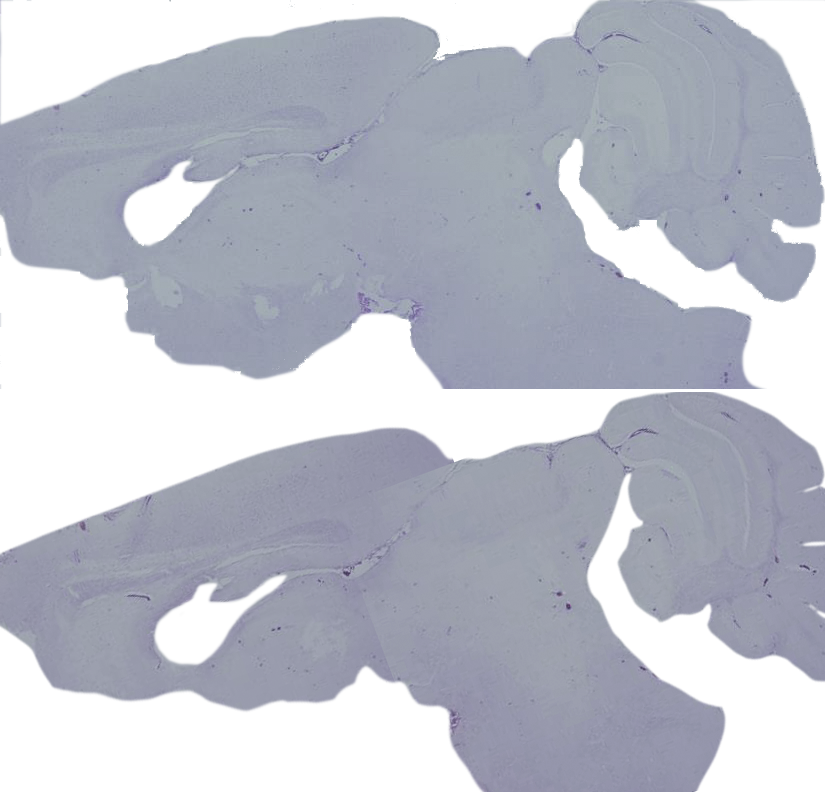


A) FUS

B) WT


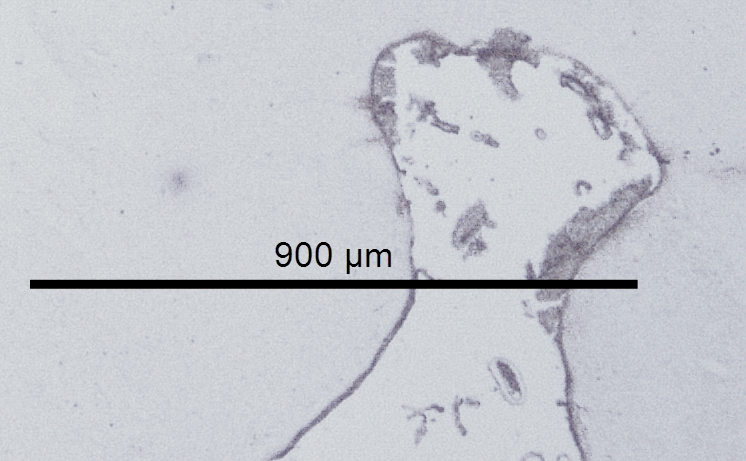


1mm

**Supplementary Figure 2. BBB status in the FUS[1-359] transgenic mice.** The photomicrographs are representative of immunohistochemistry for mouse IgG in the animals killed at 3 month of age at the time they developed clinical signs. 10um-thick paraffin sagittal sections were cut through the brains (ML+0.5mm) and mounted on gelatinized slides, which were then rehydrated to stain. The presence of IgG in the brain, as a marker of BBB dysfunction, was detected with a biotinylated horse anti-mouse IgG (H+L) (BA-2000 Vector Laboratories 1:1000) after an incubation for 24h at 4deg C in 10% normal horse serum blocking. Positivity was identified using standard ABC (Vector Laboratories, Peterborough, UK), and immunoreactivity was revealed with DAB. All sections were processed, immunolabelled and assessed for BBB breakdown in the same batch. Sections were then lightly counterstained with cresyl violet. There was no evidence of IgG extravasation in the WT control animals or the 3-month-old FUS mutants. The choroid plexus, serving as a positive control, was positively stained for IgG as expected. Thus the BBB is intact in these animals at this stage in the pathogenesis.
